# On Vaccine Inoculation

**Published:** 1810-02

**Authors:** 


### *To the Editors of the Medical and Physical Journal.*

GENTLEMEN,

WHEN the discovery of Vaccination was first promulgated by Dr. Jenner, and very soon afterwards adopted by Drs. Pearson and Woodville in the metropolis, you became its zealous promoters. The first material check given to its progress, arose from the place where Dr. Woodville chose to conduct his experiments. The lymph sent into the country from the Small-pox Hospital, produced the small-pox, or a disease so like it, that the most candid practitioners who employed it, exclaimed, "What do we gain by this new practice?" And if the error had not soon been detected and explained, it would have proved fatal to the discovery. Soon after this, every one who could obtain lymph began to insert it into the arms of the young and old, who were believed to be in danger from the infection of small-pox: and so easy and safe did the practice appear, that no one hesitated to become a vaccinator, or vaccinatrix, for sex and age were not distinguished on this occasion. This was a far more dangerous rock than that on which Dr. Woodville split; for it was with reason apprehended, that cases of failure would not be less numerous than the successful ones. When variolous inoculation was first introduced into this country, the desire of gain, *auri sacra fames,* or at least a desire to *share* in the spoil, induced every one who could procure the means, to become an inoculator; and the consequence, as might naturally be expected, were frequent failures, either in the promised mildness of the disease, or the future protection of the subjects. This new practice, however, was never presumed to be undertaken by any but such as had received some medical education; whereas vaccination, on the contrary, was supposed by many, who undertook to perform

 it, it, to require no instruction or knowledge whatever. Notwithstanding all these disadvantages, the real failures were comparatively but very few. In this period of progress, an event occurred which might very naturally have been expected; for what happened at Ephesus when St. Paul's preaching began to threaten the craft of the priests of Diana, took place here. As soon as the success of vaccination began to be established, and the tide of popular opinion to turn in its favour, a number of adversaries presented themselves, who did not oppose cool reasoning, or physiological analogies to the new practice, but on the contrary, attempted to work upon the ignorance and prejudices of the lower orders by misrepresentation.

I always admired the concise and pointed observation of Dr. Denman, on the ground which they took to rest their opposition. "All the wit, and all the argument advanced against vaccination, is contained in the word *bestial*."

While, however, I allow that the opposition to Dr. Jenner's discovery was not conducted on physiological, philosophical, or liberal principles, I must still think that your zeal in defence of it, too often induced you to admit harsh or personal observations into your Journal, in reply to those opponents.

No one will deny, that it is very natural for those who have laboured much to bring any discovery, or even produce of nature, to its utmost degree of perfection, to become jealous and irritable, when they think it rudely and improperly assailed; but I cannot admit this as a sufficient defence of your too evident partiality. If your present impartiality should induce you to admit these strictures on this part of your conduct, I may hope you will not object to what follows.

When the alleged cases of failure had been investigated, and were discovered (with very few exceptions) to be misrepresentations, or wilful falshoods; and when public opposition had nearly exhausted itself in the metropolis, the *only* spot in the world, as I am assured, where it acquired any thing like the appearance of force; his Majesty commanded the College of Physicians to investigate the subject, and make their report. The time and attention bestowed on this inquiry, gave it a weight and authority which appears to have silenced all opposition.

I can hardly abstain from expressing my regret at this event, for what before was public and avowed, became secret, or you took advantage of it, to shut the door against, not only all future *controversies* on the subject, but also, as I sup-

I suppose, all future reports and histories, whether favourable or otherwise. If you have actually taken such a resolution, in consequence of vaccination being joyfully and generally adopted in every part of the world; or from any other cause, you will at least permit me to remonstrate against the line of conduct you at present pursue. After continuing for ten years to support, cherish, and defend vaccination, quo jure quâque injuriâ, the moment it is able to go alone, or has acquired powerful protectors, you instantly renounce all care and concern about it, as if you had never laboured to shelter it from the storms by which it had been assailed.

You, who reside in the metropolis, may perhaps receive frequent information respecting the progress of a practice, from which so much was expected, or may perhaps now be realizing; but we who inhabit the distant parts of the kingdom, cannot easily forego the gratification we used to receive from your Journal, on the success attending, or impediments opposed to a discovery of such general concern. We are particularly anxious to be informed whether public opposition to vaccination has really ceased, or is only passed over without notice by you, as unworthy your attention? Whether the zeal in the cause, so conspicuous a year or two ago, still continues unabated? or whether, like the foot-ball, it remains in perfect rest, because no one thinks it worth his while to contend about it? Whether any number of well authenticated failures has damped the ardour of its supporters? or whether government is taking any active steps to propagate and support the discovery? Whether any secret and insidious means are employed to discourage parents from resorting to this mild preventive of a severe and often fatal disease? or, on the contrary, the clergy are advised by proper authority to recommend it on all occasions?

These, Gentlemen, are the leading points on which I and several others in this part of the island are anxious to receive some information; and as we know no channel through which we have a greater right to expect it, or probability of receiving it, than the medium of your Journal, I have ventured to trespass so long on the patience of yourselves, and, if you indulge me with its insertion, that of your readers, very many of whom I may suppose to have the same desire of information on this interesting subject as myself.

I am, &c.

PERCONTATOR.

*Aberdeen, Dec. 20,* 1809.

In answer to this Corerspondent, and others who may wish for similar information, the Editors assure them, that they have no intention to desert their favourite subject of Vaccination. For reasons already given in our Journal, communications respecting it will be much less frequent than heretofore, because the necessity for them no longer exists. Every thing either new or interesting shall be given with our usual zeal and promptitude, whether respecting the success, general adoption, opposition, or measures adopted by Government respecting it; and we hope the following document, which we have just received, will be a sufficient proof that vaccination is by no means in a state of inactivity. We are also assured, from the same authority, that very little reluctance against the adoption of vaccination appears in any part of the kingdom, except the metropolis. Printed instructions are given to all medical men, who apply for them, at the Board Room of the National Vaccine Establishment, respecting the progress and appearance of the *regular* vaccine vesicle; varieties not preventing the success of vaccination; irregularities and imperfections which denote the insecurity of the patient; the best manner of vaccinating, and some other subjects of less importance.

*COPY of a circular LETTER addressed by the BOARD, appointed by His MAJESTY's Government, to regulate the Affairs of the National Vaccine Establishment.*

To the Governors, Managers, Directors, and Medical Officers of the several public Charities for Assisting the indigent Sick in the Metropolis, and Kingdom at large.

*National Vaccine Establishment, Leicester Square, Dec.* 10, 1809.

"My Lords and Gentlemen,

"His Majesty's government, under the direction of Parliament, having appointed a National Vaccine Establishment, "for the purpose of rendering vaccine inoculation generally beneficial to his Majesty's subjects;' the Board thereof, consisting of the President and Censors of the Royal College of Physicians, and of the Master and Governors of the Royal College of Surgeons in London, anxious to promote his Majesty's benevolent intentions, most earnestly invite your aid and concurrence in the furtherance of this important object; under the conviction, that the sanction of your approval and snpport will have much weight upon the minds of the lower orders of the community. They are persuaded, that your influence may be successfully successfully applied, so as to effect in a considerable degree the removal of popular prejudices against vaccination, and to favour its progress to universal adoption. The Board therefore solicit your co-operation in their undertaking, in any manner which your wisdom may suggest; but they particularly recommend to your deliberation, *the establishment of gratuitous vaccination, conducted by the medical and chirurgical officers of your institution.*

"In the event of your adopting this plan, the Board will be ready at all times, upon proper application, to transmit to your appointed medical officers, a supply of vaccine lymph; and to attend to any information relative to the cow-pox, which may be deemed worthy of communication. At the same time they beg to signify their opinion, that it would be highly desirable to be favoured with a general annual report of the number of persons vaccinated at your establishment, and of the number of failures in the protection of vaccination against the small-pox, if any such cases should occur; in order that authentic materials may be collected for the formation of further important conclusions. This correspondence with the Board must be carried on by letters addressed to Dr. HERVEY, Register of the National Vaccine Establishment; under cover to the Right Hon. Richard Ryder, one of his Majesty's principal Setcretaries of State, &c. &c. &c.

"It may not be unnecessary to observe, that a public notification of your having undertaken the gratuitous vaccination of the poor at your charity, under the auspices of his Majesty's government; and the affixing of such notice to the most conspicuous part of your edifice, would be productive of much advantage, by diffusing the knowledge of the high authority which sanctions the promotion of vaccination, and of the facility with which its benefits may be obtained by your means.

"The Board cannot conclude without again expressing their anxiety, that the proposal of this address may be deemed worthy of your favourable acceptance, and sufficiently important to be carried into immediate execution; as they conceive such assistance essential to the more general adoption of vaccination, at a time when, it must be confessed, considerable repugnance to its reception prevails among the lower orders; and when the increasing extension of the contagion of small-pox renders its antidote more necessary than ever to the public safety.

"By the desire of the Board,

"L. PEPYS, PRESIDENT."

